# Supplementary material for: Food Service Kitchen Scraps as a Source of Bioactive Phytochemicals: Disposal Survey, Optimized Extraction, Metabolomic Screening and Chemometric Evaluation
Source: Metabolites. 2023 Mar 5;13(3):386. doi: 10.3390/metabo13030386 (PMC10057048; doi:10.3390/metabo13030386)
Supplement: Supplementary file 1 [file metabolites-13-00386-s001.zip › Supplementary material_FVB_vfinal.pdf]

Food Service's kitchen scraps as a source of bioactive phytochemicals: disposal survey; optimized extraction, metabolomic screening and chemometric evaluation

Tatiana de Souza Medina[a], Carolina Thomaz dos Santos D'Almeida[a,b],Talita Pimenta do Nascimento[a,b], Joel Pimentel de Abreu[c], Vanessa Rosse de Souza[c], Diego Calandrini Kalili[a], Anderson Junger Teodoro[c], Luiz Claudio Cameron[b], Maria Gabriela Koblitz[b,d], Mariana Simões Larraz Ferreira[a,b]

[a] Laboratory of Bioactives, Food and Nutrition Graduate Program, Federal University of State of Rio de Janeiro, UNIRIO;

[b] Center of Innovation in Mass Spectrometry, Laboratory of Protein Biochemistry, UNIRIO

[c] Laboratory of Functional Food, Food and Nutrition Graduate Program, UNIRIO;

[d] Laboratory of Biotechnology, Food and Nutrition Graduate Program, UNIRIO.

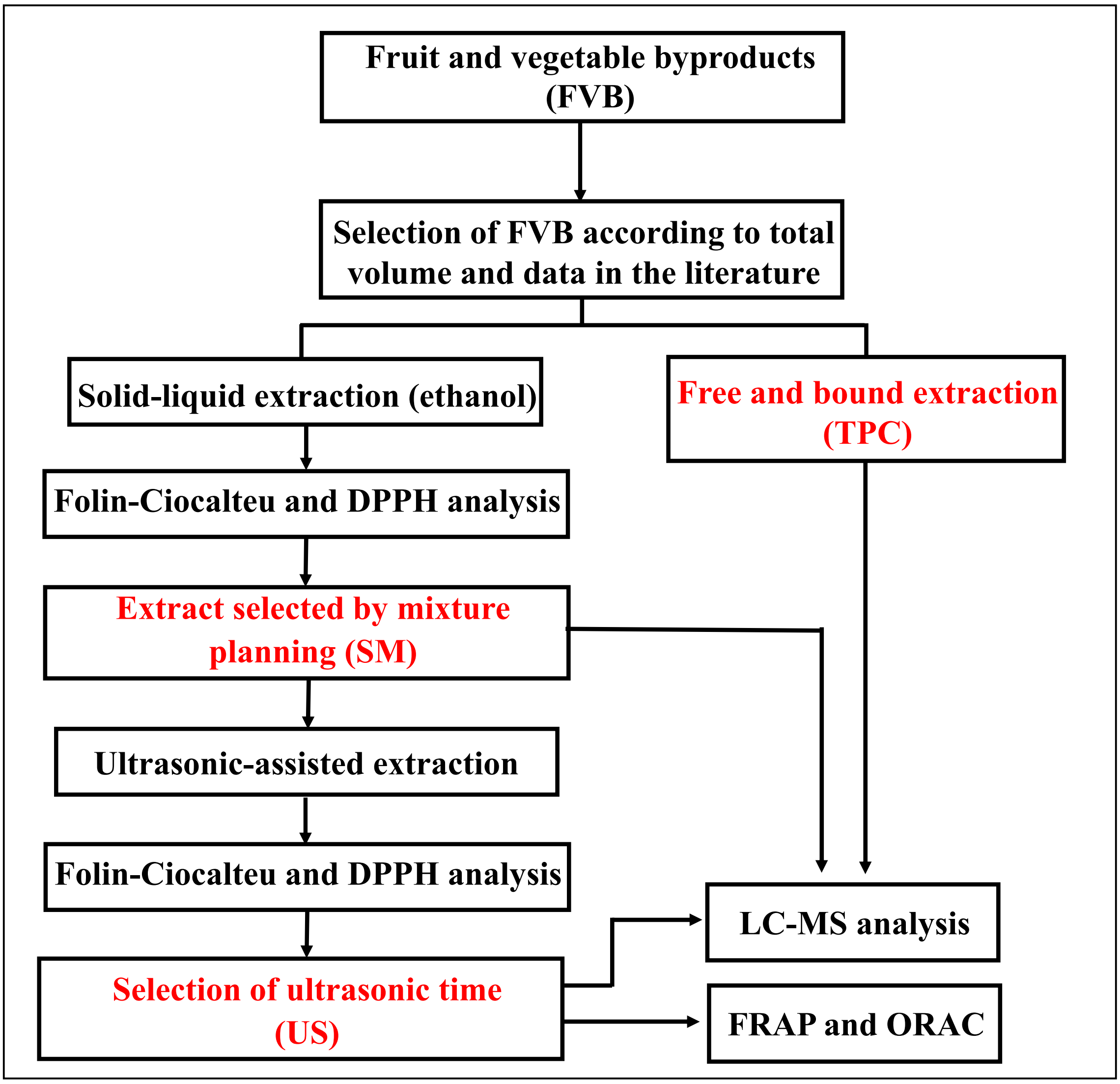

Figure S1. Flow diagram with the methodology applied for fruit and vegetable byproducts (FVB) analysis.

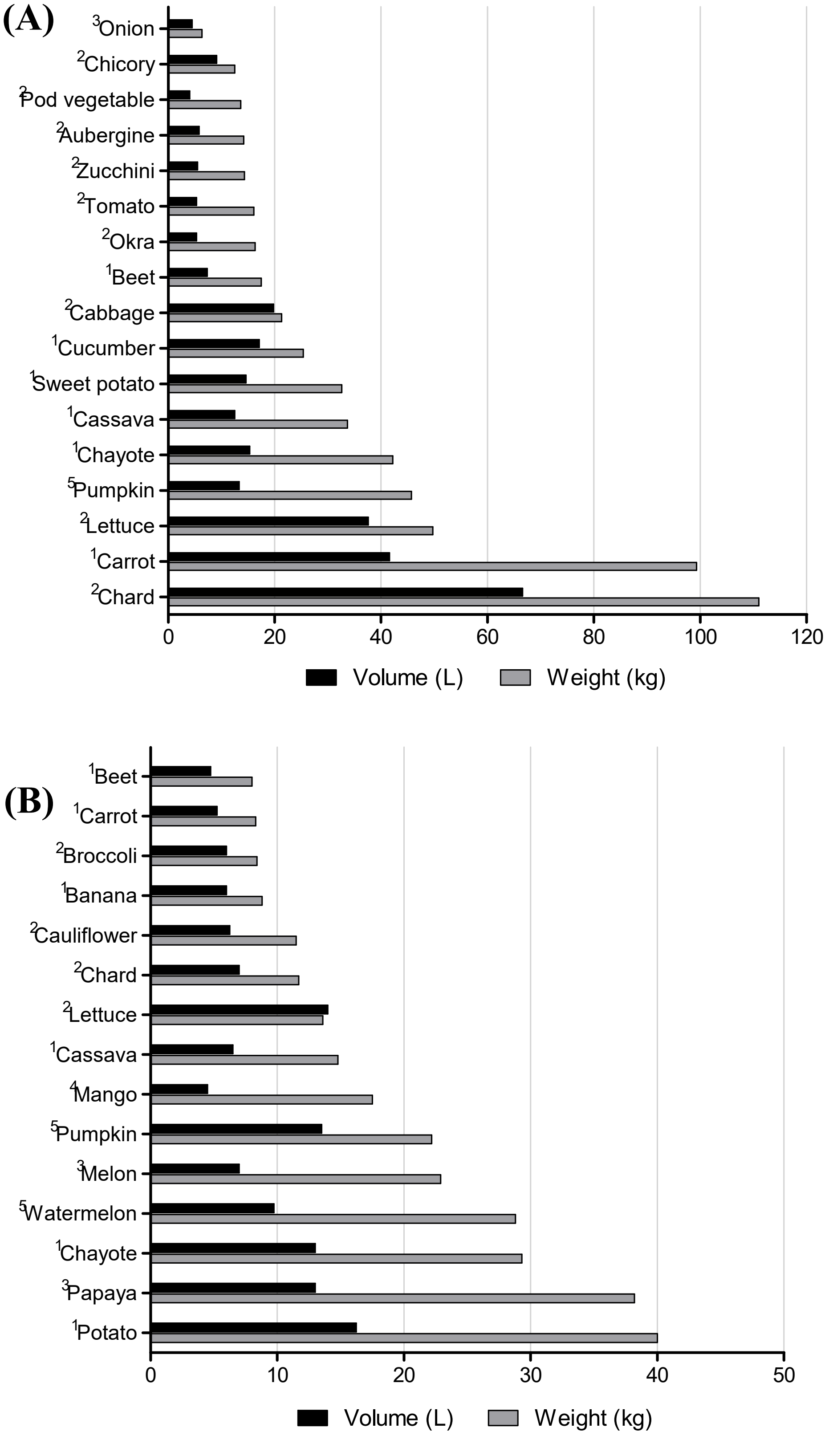

Figure S2. Survey of fruit and vegetable byproducts in weight (Kg) and volume (standard 8.4L bags), in different food services (A and B). The numbers subscripted in the names of fruits and vegetables indicate the parts where the bioproducts were obtained: 1 = peels; 2= apical part; 3= peels + apical part; 4 = peels + seeds; and 5 = peels + apical part + seeds.

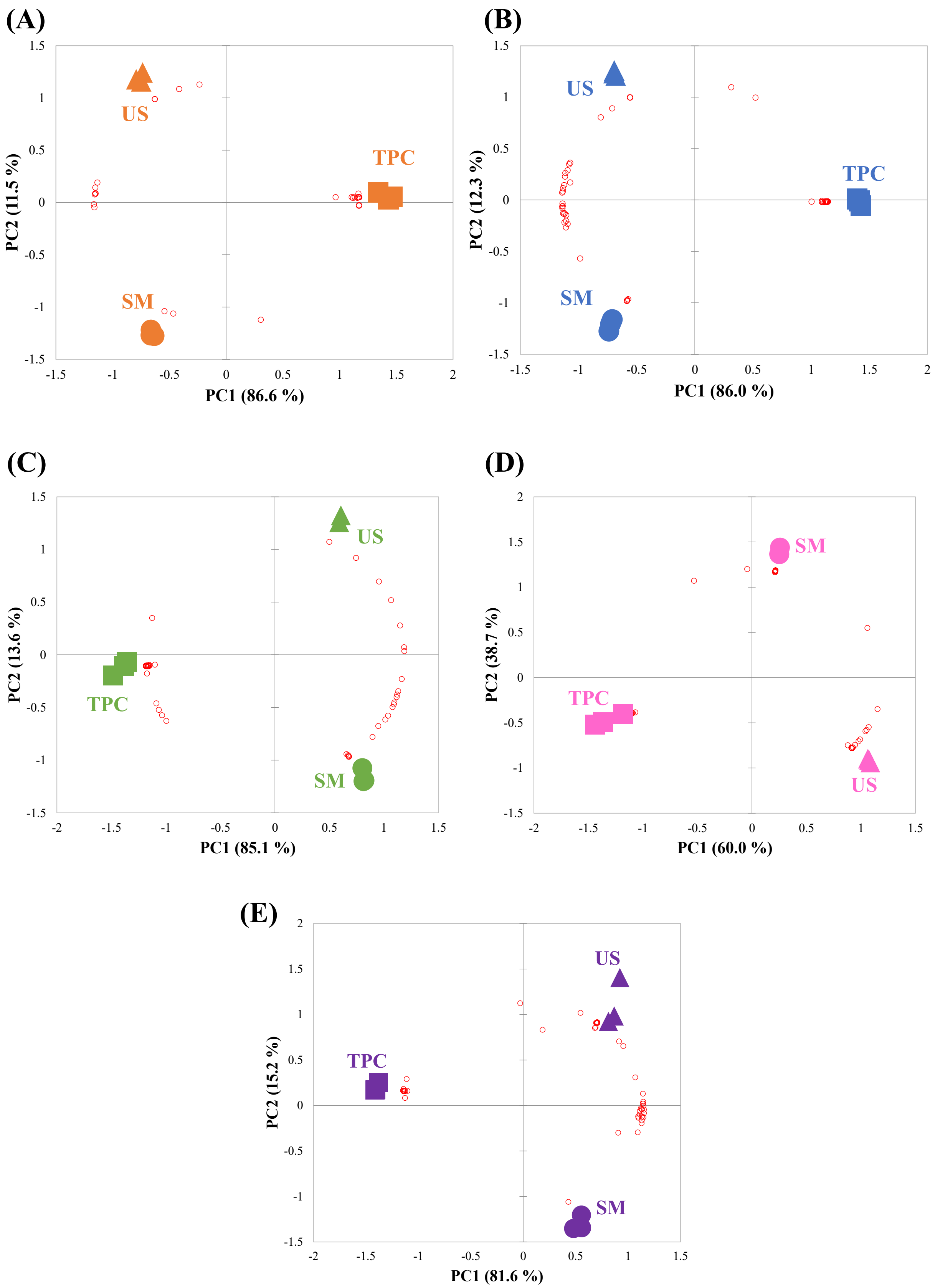

Figure S3. Principal component analysis (PCA) biplot to observe the similarity/dissimilarity between the three extracts of (A) carrot peel, (B) chard, (C) chayote peel, (D) papaya and (E) papaya seed. The samples (symbols) are distributed according to relative intensity of identified phenolic compounds (red circles).

Table S1. Total weight (kg) of five fruit and vegetable byproducts (FVB) collected in two food services, weight (g) of the dried FVB after 12h (65 ºC) and the flour yield (%) obtained after milling.

Sample	Collected FVR (kg)	Dry residue (g)	Flour Yield (%)
**Carrot peel**	1.99	212	10.70
**Chard**	2.19	104	4.80
**Chayote peel**	2.43	150	6.20
**Papaya**	2.19	259	11.80
**Papaya seed**	2.84	430	15.10

Table S2. Experimental values of total reducing content (TRC) and antioxidant capacity (AC) by DPPH of papaya, comparing extractors 75:25 and 60:40 water:ethanol.

**Extractors**	**TRC (mg GAE/100g sample flour, db)**	**AC (DPPH) (µmol TE/g sample flour, db)**
**60:40 water:etanol**	1201.95 ±20.16[a]	270.10±21.96[a]
**75:25 water:etanol**	1332.92±14.28[a]	338.30±41.15[a]

Results are expressed as mean ± standard deviation (n=3). Different letters indicate a significant difference between extractors (Tukey, $p<0.05$).

Table S3. All phenolic compounds tentatively identified by LC-MSE separated by classes and in retention time order.

Please refer to the PDF file.
