# Supplementary material for: Food Service Kitchen Scraps as a Source of Bioactive Phytochemicals: Disposal Survey, Optimized Extraction, Metabolomic Screening and Chemometric Evaluation
Source: Metabolites. 2023 Mar 5;13(3):386. doi: 10.3390/metabo13030386 (PMC10057048; doi:10.3390/metabo13030386)
Supplement: Supplementary file 1 [file metabolites-13-00386-s001.zip › Table S3_FVB_vfinal.pdf]

Table S3. All phenolic compounds tentatively identified by LC-MS^E separated by classes and in retention time order.

Number	Common Name	Molecular Formula	m/z (exp.)	RT (min)	Score (%)	Frag Score (%)	Frag database	Mass Error (ppm)	Isotope Similarity	Normalised abundance														
										TPC					SM					US				
										Carrot peel	Chard	Chayote peel	Papaya	Papaya seed	Carrot peel	Chard	Chayote peel	Papaya	Papaya seed	Carrot peel	Chard	Chayote peel	Papaya	Papaya seed
**FLAVONOIDS**																								
1	Neohesperidin	C28H34O15	609.1879	0.57	43.3	37.1	59.0120 (6.16%); 71.0120 (39.12%); 89.0226 (21.71%); 119.0330 (9.30%); 127.0380 (5.86%); 143.0331 (8.73%); 161.0437 (19.61%); 439.0833 (4.84%); 503.1611 (10.41%); 533.1717 (6.11%)	8.88	89.46						X		X	X	X			X		X
2	Theaflavin	C29H24O12	563.1220	0.62	37.6	0.2	83.0120 (10.88%); 85.0319 (5.45%)	4.47	92.76									X						
3	Rhamnetin	C16H12O7	315.0517	0.69	37.1	5.0	123.0431 (4.14%); 159.0090 (5.56%)	2.03	82.68								X					X		
4	Cyanidin 3-O-galactoside	C21H21O11+	448.1049	0.77	36.5	0.1	92.0240 (0.94%)	8.54	91.78							X	X				X	X	X	
5	Peonidin 3-O-arabinoside	C21H21O11+	448.1027	1.41	41.0	21.4	330.0770 (2.89%); 341.0922 (9.16%)	3.62	88.10							X					X		X	
6	Cyanidin 3-O-(6"-caffeoyl-glucoside)	C30H27O14+	610.1267	2.72	43.1	41.7	73.0277 (15.38%); 83.0120 (9.13%); 99.0070 (15.24%); 117.0327 (2.21%); 313.0915 (20.36%)	-9.94	84.84									X						X
7	Cirsimaritin	C17H14O6	313.07014	2.94	37.8	0	0	-5.16	95.03			X												
**8**	**(-)-Epigallocatechin**	**C15H14O7**	**305.0690**	**3.80**	**38.9**	**7.3**	**123.0432 (3.31%); 167.0331 (10.945); 197.0437 (2.28%)**	**7.47**	**95.41**	**X**	**X**		**X**	**X**	**X**	**X**		**X**	**X**	**X**	**X**		**X**	**X**
9	Kaempferol 3-O-rhamnoside	C21H19O10-	430.0918	3.88	35.5	0.0	0	2.97	80.77							X					X		X	
10	Tectoridin	C22H22O11	461.1084	3.90	37.7	0.0	0	-1.24	88.06															X
**11**	**(-)-Epicatechin**	**C15H14O6**	**289.06921**	**3.91**	**36.6**	**0**	**0**	**-8.81**	**92.51**	**X**	**X**		**X**	**X**										
12	Kaempferide	C16H11O6-	298.0499	3.94	36.0	0.0	0	5.34	85.99							X	X	X				X		
13	Chrysoeriol 7-O-apiosyl-glucoside	C27H30O15	593.1509	4.10	47.8	43.4	73.0278 (36.10%); 83.0120 (30.46%); 193.0490 (82.02%); 259.0594 (2.03%); 283.0598 (3.65%); 353.0652 (83.92%); 383.0753 (38.07%)	-0.52	96.46			X					X					X		
14	Peonidin 3-O-glucoside	C22H23O11+	462.1182	4.13	36.3	0.6	79.0171 (6.48%); 107.0120 (22.48%)	3.01	84.48									X						
15	Luteolin 6-C-glucoside	C21H20O11	447.0898	4.17	42.8	29.2	92.0250 (2.835); 117.0327 (18.70%); 130.0288 (3.03%); 145.0276 (4.72%); 232.0548 (3.25%); 273.0394 (1.17%); 401.0846 (4.24%)	-7.70	93.45			X	X			X	X				X	X	X	
16	Eriocitrin/Neoeriocitrin	C27H32O15	595.1664	4.36	36.9	0.0	0	-0.66	84.51								X					X		
17	Quercetin 3-O-rhamnosyl-rhamnosyl-glucoside	C33H40O20	755.2042	4.55	48.5	54.0	151.0017 (4.39%); 156.0404 (3.17%); 255.0282 (5.92%); 271.0232 (9.71%); 272.0286 (2.32%); 300.0259 (100%); 301.0310 (30.02%)	0.25	88.75				X					X						X
18	Apigenin 7-O-apiosyl-glucoside	C26H28O14	563.1400	4.60	51.0	62.0	117.0326 (7.03%); 267.0258 (1.76%); 282.0518 (4.75%); 283.0593 (8.76%); 297.0357 (11.04%); 309.0372 (1.30%); 311.0547 (10.09%); 323.0547 (9.63%); 324.0613 (6.21%); 325.0701 (26.84%); 353.0651 (100%); 365.0654 (18.05%); 383.0759 (70.08%); 395.0762 (5.87%); 413.0869 (6.69%); 443.0973 (12.70%); 473.1082 (8.11%)	-1.07	94.09			X					X					X		
19	Genistin	C21H20O10	431.1012	4.62	35.2	0.0	0	6.45	83.55										X					X
20	Quercetin 3-O-glucuronide	C21H18O13	477.0632	4.66	41.2	25.7	446.0454 (3.02%)	-8.84	90.24		X					X	X	X			X		X	
21	Luteolin 7-O-rutinoside	C27H30O15	593.1509	4.72	37.1	0.0	0	-0.47	85.99			X	X				X					X		
22	Dihydroformononetin	C16H14O4	269.08121	4.76	55.1	95.5	121.0274 (100%)	-2.68	83.25			X												
23	Apigenin 6,8-di-C-glucoside	C27H30O15	593.1515	4.82	37.6	0.0	0	0.45	88.36								X					X		
24	4'-O-Methylepigallocatechin	C16H16O7	319.08228	4.91	38.1	0	0	-0.14	90.86	X		X												
25	Apigenin 6-C-glucoside	C21H20O10	431.09714	4.99	56.5	93	283.0595 (78.06%); 300.0273 (100%); 301.0334 (47.81%); 311.0543 (38.98%); 359.0659 (28.68%)	-2.84	92.91			X	X											
26	Apigenin 7-O-neohesperidoside	C27H30O14	577.1556	5.01	48.7	56.4	116.0490 (84.87%); 353.0653 (18.57%)	-1.23	88.48			X	X				X					X		
27	5,7-Dihydroxyflavanone/Isoliquiritigenin/Dihydrodaidzein	C15H12O4	255.0653	5.11	36.0	0.0	0	-3.76	84.17															X
28	Not id compound I	C16H12O6	299.05583	5.18	38.6	0	0	-0.95	94.22		X			X										
29	Quercetin 3-O-rutinoside	C27H30O16	609.1461	5.24	48.2	44.3	255.0284 (7.89%); 263.0582 (100%); 271.0232 (21.35%); 301.0326 (42.46%)	0.00	96.89				X					X						
30	Pebrellin	C19H18O8	373.0950	5.38	37.4	0.0	0	5.76	93.81									X						
31	Gardenin B	C19H18O7	357.0969	5.43	36.1	0.0	0	-3.12	83.93									X						
32	Apigenin 7-O-rutinoside	C27H30O14	577.1561	5.46	44.0	32.7	59.0118 (27.31%); 158.0593 (100%); 353.0651 (89.60%)	-0.32	87.79								X					X		
33	Eriodictyol	C15H12O6	287.0548	5.47	42.0	18.4	59.0118 (32.92%); 241.0493 (46.14%)	-4.41	96.51		X			X										X
34	Phloretin	C15H14O5	273.0755	5.49	41.0	26.4	241.0493 (43.98%)	-4.84	83.95		X			X					X					X
35	Glycitin	C22H22O10	445.1133	5.53	39.0	8.1	117.0331 (17.47%); 309.0611 (1.90%)	-1.52	88.68							X					X		X	
36	Eupatorin/Cirsilineol	C18H16O7	343.0847	5.63	36.9	0.0	0	6.79	91.98										X					X
37	Not id compounds II	C16H12O6	299.05569	5.63	57.6	97.7	269.0443 (100%)	-1.39	91.95		X													

38	6-Hydroxyluteolin	C15H10O7	301.0340	5.67	53.1	87.2	283.0234 (27.33%); 284.0308 (14.02%); 285.0308 (100%)	-4.48	83.59		X													X
39	7,8,4'-Trihydroxyisoflavone	C15H10O5	269.0475	5.73	39.6	9.5	185.0229 (13.30%); 186.0303 (19.45%)	7.08	96.37										X					X
40	Irilone	C16H10O6	297.04016	5.78	36.3	0	0	-1.01	82.89		X	X	X	X										
41	Neodiosmin	C28H32O15	607.16841	5.78	57.3	98.9	299.0556 (100%)	2.57	90.79			X	X											
42	Dihydroquercetin	C15H12O7	303.0500	5.85	38.4	12.8	269.0444 (9.53%)	-3.54	83.57															X
43	Myricetin 3-O-glucoside	C21H20O13	479.0828	5.93	42.6	16.7	119.0122 (6.21%); 144.0443 (84.67%); 163.0027 (7.34%); 181.0129 (4.02%); 299.0159 (4.24%)	-0.58	97.15		X					X					X		X	
44	Tetrahydroxyisoflavone isomer	C15H10O6	285.03934	6.10	39.8	11.2	108.0210 (10.26%); 190.0274 (17.28%)	-3.90	92.25		X			X										
45	Diosmetin 7-O-rutinoside	C28H32O15	607.1665	6.14	38.3	3.6	153.0175 (4.47%)	-0.50	88.71			X	X				X							
**46**	**Kaempferol**	**C15H10O6**	**285.0391**	**6.40**	**38.4**	**0.0**	**0**	**-4.87**	**97.40**			**X**	**X**						**X**					**X**
47	3,7-Dimethylquercetin	C17H14O7	329.06626	6.48	39.8	18.8	193.0495 (19.36%)	-1.26	81.78			X												
48	Irisolidone	C17H14O6	313.07145	6.51	38.5	0	0	-0.99	93.72			X												
49	Not id compound III	C16H14O5	285.07688	6.74	36.6	0	0	0.13	83.05			X												
50	Jaceosidin	C17H14O7	329.0671	6.76	36	0	0	1.29	81.78			X												
51	Quercetin 3-O-galactoside	C21H20O12	463.0877	6.77	46.1	35.0	141.0174 (34.28%); 365.0501 (4.71%); 379.0659 (15.90%)	-1.04	96.76							X					X		X	
52	Homoeriodictyol	C16H14O6	301.0707	6.78	35.8	0.0	0	-3.40	82.85		X									X				
53	Butein	C15H12O5	271.05955	6.92	40.2	21.6	134.0360 (69.20%); 135.0450 (100%)	-6.04	86.08		X	X												
54	6''-O-Malonylglycitin	C25H24O13	531.1145	7.04	39.4	11.8	296.0676 (2.94%); 311.0920 (2.83%)	0.12	85.56							X								
55	Nobiletin	C21H22O8	401.1233	7.05	39.0	5.5	176.0465 (18.60%)	-2.11	91.97							X					X		X	
56	Not id compound IV	C15H12O5	271.0599	7.11	36.6	0.0	0	-4.90	88.50	X									X					X
57	O-Desmethylangolensin	C15H14O4	257.08187	7.24	38	0	0	-0.25	90.18		X													
58	Tetramethylscutellarein	C19H18O6	341.1027	7.48	37.7	0.0	0	-1.08	89.67			X					X				X		X	
59	Formononetin	C16H12O4	267.0654	7.55	41.2	20.1	92.0263 (100%)	-3.32	89.95							X					X		X	
60	Sakuranetin	C16H14O5	285.0757	7.83	45.7	50.1	133.0274 (11.57%); 134.0352 (42.47%); 149.0589 (8.66%); 178.0249 (3.22%); 193.0489 (100%)	-4.11	83.05											X				
61	Quercetin	C15H10O7	301.0341	7.86	45.3	36.0	65.0014 (2.52%); 107.0117 (15.08%); 151.0016 (25.10%)	-4.16	95.60									X	X					X
62	Hesperetin	C16H14O6	301.0704	7.91	54.0	91.1	92.0249 (100%)	-4.38	84.12		X													X
63	Violanone	C17H16O6	315.0860	7.93	56.0	95.9	92.0249 (100%)	-4.36	89.21	X										X				
64	(+)-Catechin	C15H14O6	289.0705	8.31	36.5	0.0	0	-4.42	87.69										X					X
65	Not id compound V	C15H12O5	271.0598	8.44	35.6	0.0	0	-4.97	83.97															X
66	Trihydroxyflavone isomer	C15H10O5	269.04459	8.55	56.8	95	117.0338 (100%)	-3.54	93.10			X	X											
67	Apigenin	C15H10O5	269.0440	8.89	39.5	8.2	84.0205 (8.01%); 106.0410 (13.11%); 107.0119 (13.24%); 117.0327 (100%); 151.0018 (12.01%); 225.0532 (1.66%); 237.0579 (5.72%)	-5.82	96.13								X					X		
68	Quercetin 3-O-glucoside	C21H20O12	463.0861	8.94	55.4	96.0	255.0289 (11.84%); 271.0236 (25.01%); 300.0268 (100%); 301.0334 (82.74%)	-4.58	86.41							X					X		X	
**69**	**Myricetin**	**C15H10O8**	**317.0289**	**8.95**	**35.7**	**0.0**	**0**	**-4.26**	**83.39**		**X**								**X**					**X**
70	Hispidulin	C16H12O6	299.0545	9.21	50.1	59.2	63.0222 (3.82%); 83.0121 (10.50%); 132.0197 (4.29%); 133.0275 (12.79%); 134.0349 (8.58%); 146.0352 (1.15%); 148.0143 (2.23%); 187.0381 (2.58%); 199.0382 (6.67%); 200.0452 (3.88%); 211.0382 (8.75%); 227.0332 (20.71%); 239.0335 (1.51%); 255.0283 (8.29%); 256.0359 (21.05%); 284.0311 (100%)	-5.43	97.33			X	X				X					X		
71	Nepetin/Isorhamnetin	C16H12O7	315.0495	9.29	36.8	0.0	0	-4.69	89.38									X						
72	3'-O-Methylviolanone	C18H18O6	329.1016	10.39	45.5	51.3	84.0196 (46.89%); 136.0148 (100%)	-4.35	81.06															X
73	Dihydroxyflavone isomer	C15H10O4	253.0487	11.35	35.2	0.0	0	-7.42	84.19								X							
74	Naringenin O-glucoside	C21H22O10	433.1107	11.86	33.8	0.0	0	-7.60	77.72								X							
	**PHENOLIC ACIDS**																							
75	Ellagic acid	C14H6O8	300.9991	0.85	36.8	0.0	0	0.46	84.33										X					X
76	Hydroxyhippuric acid	C9H9NO4	194.0449	1.63	38.8	5.6	120.0107 (7.97%)	-5.23	94.26							X		X		X	X		X	
77	Dihydroxybenzoic acid isomer I	C7H6O4	153.01878	1.78	56.4	90.3	138.0314 (100%)	-3.59	95.79	X	X	X	X	X										
78	Dihydroxybenzoic acid isomer II	C7H6O4	153.01945	1.94	39.2	0.0	0	0.77	97.18	X	X	X	X	X										
**79**	**Vanillic acid**	**C8H8O4**	**167.0342**	**2.03**	**53.0**	**72.7**	**150.0308 (77.18%); 151.0387 (100%)**	**-4.57**	**97.69**	**X**	**X**	**X**	**X**	**X**										
80	Dihydroxybenzoic acid isomer III	C7H6O4	153.01876	2.24	56.9	90.9	108.0210 (35.36%); 109.0286 (100%)	-3.70	97.76	X	X	X	X	X										
81	O-Methylgallic acid isomer I	C8H8O5	183.0287	2.35	40.8	18.6	149.9941 (1.52%); 168.0047 (2.43%)	-6.65	92.95			X				X					X		X	
82	Caffeoylquinic acid isomer I	C16H18O9	353.08748	2.46	52.1	65.2	191.0562 (59.45%)	-0.93	96.54	X	X													
**83**	**Gallic acid**	**C7H6O5**	**169.0133**	**2.50**	**48.4**	**49.2**	**123.0068 (100%)**	**-5.32**	**99.03**		**X**	**X**	**X**	**X**					**X**					**X**
84	1-Caffeoylquinic acid	C16H18O9	353.0871	2.57	41.6	17.3	219.0283 (96.91%)	-1.86	93.09												X		X	
85	Gallic acid 3-O-gallate	C14H10O9	321.0276	2.62	37.4	0.0	0	7.50	95.25							X					X		X	
86	Dihydrocaffeic acid	C9H10O4	181.05006	2.67	37.8	0.0	0	-3.12	92.62			X	X											
87	Caffeoylquinic acid isomer II	C16H18O9	353.0870	2.68	42.8	21.6	321.0271 (1.64%)	-2.41	95.10						X			X		X	X		X	X
88	5-O-Galloylquinic acid	C14H16O10	343.0658	2.76	43.5	26.3	101.0225 (100%); 161.0437 (37.01%); 167.0337 (10.79%), 205.0122 (0.54%)	-3.69	95.57										X					X
89	Homoveratric acid	C10H12O4	195.0651	2.87	36.4	0.0	0	-6.03	88.81		X													
90	p-Coumaric acid 4-O-glucoside	C15H18O8	325.0952	2.90	39.1	7.1	161.0225 (62.13%)	7.14	96.41	X	X					X					X		X	
**91**	**3,4-Dihydroxyphenylacetic acid**	**C8H8O4**	**167.0352**	**2.93**	**58.6**	**96.1**	**108.0217 (93.03%); 123.0455 (100%)**	**1.13**	**98.08**	**X**	**X**			**X**										
92	4-Hydroxyphenylacetic acid	C8H8O3	151.03911	3.06	56.5	95.7	135.0453 (100%)	-6.29	94.04		X	X	X	X										
**93**	**2,5-Dihydroxybenzoic acid**	**C7H6O4**	**153.0185**	**3.10**	**38.6**	**0.0**	**0**	**-5.27**	**99.01**		**X**	**X**	**X**	**X**										

**94**	**Benzoic acid**	**C7H6O2**	**121.0285**	**3.35**	**38.0**	**0.0**	**0**	**-8.12**	**99.23**	**X**	**X**	**X**	**X**	**X**										
**95**	**5-Caffeoylquinic acid**	**C16H18O9**	**353.0864**	**3.41**	**45.1**	**33.3**	**85.0276 (4.53%); 87.0069 (1.29%); 127.0381 (1.82%); 135.0431 (1.83%); 161.0224 (2.50%); 173.0437 (1.26%); 191.0543 (100%)**	**-3.91**	**96.87**	**X**	**X**				**X**	**X**		**X**	**X**	**X**	**X**	**X**	**X**	**X**
96	4-Hydroxymandelic acid	C8H8O4	167.03415	3.42	55.5	92.1	125.0244 (100%)	-4.98	91.19		X			X										
97	3-Hydroxyphenylacetic acid	C8H8O3	151.03887	3.44	52.1	77.0	125.0244 (100%)	-7.89	92.06		X	X	X	X										
98	Vanillactic acid	C10H12O5	211.0594	3.51	41.8	25.9	168.0768 (35.97%)	-8.47	92.41				X		X					X				
99	3-Feruloylquinic acid	C17H20O9	367.1025	3.59	45.4	31.2	117.0332 (18.38%); 155.0329 (12.09%); 193.0493 (100%)	-2.69	98.92	X	X				X	X				X	X		X	
100	Phenylacetic acid	C8H8O2	135.0448	3.60	39.3	0.0	0	-2.55	99.76	X	X	X	X	X										
101	Dihydroxybenzoic acid isomer IV	C7H6O4	153.0189	3.64	39.1	0.0	0	-3.04	98.92		X	X	X	X										
102	Feruloyl glucose	C16H20O9	355.1026	3.64	35.9	0.0	0	-2.40	82.21	X	X													
103	2,3,4-Trihydroxybenzoic acid	C7H6O5	169.01409	3.65	47.5	44.9	113.0239 (60.82%)	-0.91	93.55		X			X										
104	Phenacetylglycine	C10H11NO3	192.0648	3.72	37.6	0.0	0	-9.48	98.31									X	X					X
105	Caffeoylquinic acid isomer III	C16H18O9	353.08644	3.75	56.6	98.1	191.0562 (100%)	-3.86	89.62	X														
106	Dihydroferulic acid	C10H12O4	195.0646	3.86	36.4	4.8	73.0277 (100%); 83.0120 (76.95%)	-8.40	86.50		X						X					X		
107	4-p-Coumaroylquinic acid	C16H18O8	337.09195	3.92	57.9	98.0	119.0508 (24.03%); 191.0561 (100%)	-2.77	94.52	X	X													
108	Homovanillic acid	C9H10O4	181.05063	3.96	38.2	0.0	0	0.00	90.88	X														
109	O-Methylgallic acid isomer II	C8H8O5	183.0281	4.20	37.5	0.0	0	-10.00	98.50		X								X					X
110	m-Coumaric acid	C9H8O3	163.03939	4.31	37.3	0.0	0	-4.11	91.24	X		X	X											
111	Rosmarinic acid	C18H16O8	359.0792	4.32	38.5	14.2	135.0431 (100%); 239.0548 (34.16%)	5.49	84.62	X					X									
**112**	**p-Coumaric acid**	**C9H8O3**	**163.03972**	**4.58**	**58.1**	**96.2**	**119.0510 (100%)**	**-2.15**	**96.94**	**X**	**X**	**X**	**X**	**X**										
**113**	**Cinnamic acid**	**C9H8O2**	**147.0452**	**4.62**	**39.7**	**0.0**	**0**	**0.35**	**98.71**	**X**		**X**	**X**	**X**										
114	Ethyl caffeate	C11H12O4	207.06478	4.69	35.9	0.0	0	-7.21	87.87		X	X												
**115**	**Isoferulic acid**	**C10H10O4**	**193.05006**	**5.03**	**57.0**	**90.2**	**130.0422 (100%); 133.0286 (25.06%)**	**-2.95**	**98.48**	**X**	**X**	**X**	**X**	**X**										
116	Kaempferol 3-O-rutinoside	C27H30O15	593.1509	5.26	37.0	4.7	283.0234 (4.97%)	-0.43	80.70								X							
117	Trans-ferulic acid	C10H10O4	193.0489	5.36	37.6	0.0	0	-8.75	97.52							X	X	X			X	X	X	
118	Dicaffeoylquinic acid isomer I	C25H24O12	515.1199	5.51	42.8	24.1	161.0224 (22.64%)	0.78	90.98	X														
119	Ferulic acid	C10H10O4	193.0490	5.53	42.0	21.7	117.0331 (100%)	-8.59	97.67	X	X	X	X	X		X	X	X	X		X	X	X	X
**120**	**Sinapic acid**	**C11H12O5**	**223.0598**	**5.56**	**46.1**	**40.2**	**121.0282 (66.82%); 149.0230 (100%); 166.0253 (5.33%); 167.0330 (54.78%)**	**-6.35**	**97.63**		**X**		**X**			**X**		**X**			**X**		**X**	
121	Dicaffeoylquinic acid isomer II	C25H24O12	515.1200	5.66	41.1	8.2	161.0234 (3.54%)	0.90	98.21	X														
122	Hippuric acid	C9H9NO3	178.0497	5.68	35.4	0.0	0	-7.12	85.15							X								
123	Dihydro-p-coumaric acid	C9H10O3	165.05481	5.90	38.3	0.0	0	-5.45	97.53	X														
**124**	**4-Hydroxybenzoic acid**	**C7H6O3**	**137.0238**	**5.92**	**50.5**	**58.1**	**92.0257 (80.05%); 119.0122 (100%)**	**-4.30**	**99.49**	**X**	**X**	**X**	**X**	**X**	**X**				**X**					**X**
125	Dicaffeoylquinic acid isomer III	C25H24O12	515.1199	6.03	50.7	56.8	135.0446 (59.78%); 179.0346 (73.43%); 353.0872 (5.26%)	0.74	97.37	X														
126	Dicaffeoylquinic acid isomer IV	C25H24O12	515.1186	6.29	44.1	25.0	135.0431 (41.76%); 179.0330 (67.93%); 353.0865 (9.38%)	-1.77	97.62						X					X				
127	Danshensu	C9H10O5	197.0457	7.16	40.6	14.4	141.0542 (7.46%); 144.0440 (15.20%); 154.0642 (10.18%); 156.0439 (19.05%); 172.0392 (7.65%)	0.76	89.58							X								
**128**	**Caffeic acid**	**C9H8O4**	**179.0346**	**7.67**	**56.8**	**87.7**	**135.0445 (100%)**	**-1.94**	**98.56**	**X**	**X**	**X**	**X**	**X**	**X**									
129	Cinnamoyl glucose	C15H18O7	309.1004	8.87	37.1	10.9	85.0281 (39.49%); 265.0528 (5.77%); 267.0901 (17.29%); 295.0854 (9.94%)	7.78	83.51							X								
**OTHER POLYPHENOLS**																								
130	Hydroxytyrosol	C8H10O3	153.05473	1.08	36.7	0	0	-6.42	91.04					X										
131	4-Vinylphenol	C8H8O	119.04967	1.10	37.2	0	0	-4.73	91.47		X		X	X										
132	Oleuropein	C25H32O13	539.1718	1.12	38.3	8.8	118.0284 (0.82%); 134.0593 (32.88%); 175.0400 (1.56%); 206.0447 (2.69%); 212.0668 (2.23%); 242.0769 (3.12%)	-9.58	93.05									X						
133	Curcumin	C21H20O6	367.1162	1.24	36.7	3.1	83.0120 (4.58%); 97.0277 (17.17%)	-6.82	87.90									X						
134	3,4-Dihydroxyphenylglycol	C8H10O4	169.0495	1.67	37.9	0.0	0	-6.52	96.67												X		X	
135	Catechol	C6H6O2	109.02891	1.78	38.1	0	0	-5.42	96.82		X			X										
136	Salvianolic acid D	C11H10O6	237.0391	2.23	44.4	35.9	181.0123 (100%)	-5.57	92.34										X					X
**137**	**Pyrogallol**	**C6H6O3**	**125.0235**	**2.50**	**38.4**	**2.8**	**79.0185 (3.68%)**	**-7.42**	**97.71**		**X**	**X**	**X**	**X**					**X**					**X**
138	Methylcatechol isomer I	C7H8O2	123.04527	2.51	39.6	0	0	0.92	99.21		X			X										
139	Syringaldehyde	C9H10O4	181.0489	2.92	38.5	12.0	116.0488 (100%)	-9.61	91.14	X		X				X	X	X	X	X	X	X	X	X
140	Protocatechuic aldehyde	C7H6O3	137.0237	2.93	55.2	83.6	108.0217 (93.03%); 123.0455 (100%)	-5.47	98.84	X	X	X	X	X										
141	Resacetophenone	C8H8O3	151.03886	2.94	55.5	91.5	108.0217 (93.03%); 123.0455 (100%)	-7.95	94.78			X												
142	Acetyl eugenol	C12H14O3	205.0866	2.94	37.3	0.0	0	-2.04	88.88								X							
143	Isopimpinellin	C13H10O5	245.04432	3.05	36	0	0	-4.98	85.81			X												
144	Myrtenic Acid	C9H6O3	161.02443	3.19	50.9	57.5	133.0297 (40.60%); 134.0368 (15.40%); 135.0450 (67.22%)	0.10	97.03	X														
145	Methylcatechol isomer II	C7H8O2	123.04432	3.26	51.8	69	108.0214 (100%)	-6.69	97.84		X			X										
146	5-Tricosylresorcinol	C29H52O2	431.3867	3.26	36.90	8.46	109.0272 (64.72%); 119.0487 (100%)	-6.33	83.42						X	X				X				
147	Oleoside dimethylester	C18H26O11	417.1398	3.32	38.4	0.0	0	-0.93	93.26									X						
148	[6]-Gingerol	C17H32O4	299.2204	3.36	34.7	0.0	0	-7.97	82.19										X					X
149	Esculetin	C9H6O4	177.01857	3.54	38.1	0	0	-4.26	95.69	X	X	X	X	X										
150	6,7-Furanocoumarin	C11H6O3	185.0259	3.54	37.6	8.7	145.0278 (10.48%)	7.74	88.21										X					
151	Coumarin	C9H6O2	145.0306	3.60	40.0	16.3	117.0332 (57.38%)	7.33	92.09	X	X			X	X				X					X
152	Scopoletin	C10H8O4	191.0332	3.60	40.7	14.3	97.0276 (15.77%); 99.0432 (33.71%)	-9.36	99.45			X			X				X	X				X
153	Arbutin	C12H16O7	271.0812	3.91	39.8	6.0	141.0170 (0.74%)	-4.10	97.71		X					X					X		X	

154	Phenol	C6H6O	93.035067	3.94	38.5	0	0	5.09	98.56		X	X		X										
155	Umbelliferone	C9H6O3	161.02442	3.96	39.3	0	0	0.01	96.61	X	X			X										
**156**	**Vanillin**	**C8H8O3**	**151.03954**	**4.46**	**38**	**0**	**0**	**-3.47**	**93.86**	**X**	**X**	**X**												
157	Ferulaldehyde/Mellein	C10H10O3	177.0564	4.49	37.7	0	0	3.83	93.22		X													
158	Esculin	C15H16O9	339.07091	5.28	48.3	50.8	121.0289 (100%); 149.0237 (84.01%)	-3.67	95.21		X													
159	Sinapaldehyde	C11H12O4	207.06658	5.90	38.3	0	0	1.44	93.11	X														
160	Coumestrol	C15H8O5	267.0319	6.45	35.1	0.0	0	7.64	84.01										X					X
**161**	**4-Hydroxybenzaldehyde**	**C7H6O2**	**121.0290**	**7.55**	**57.8**	**94.9**	**92.0263 (100%)**	**-3.91**	**98.89**	**X**	**X**	**X**	**X**	**X**										**X**
162	Demethoxycurcumin	C20H18O5	337.10822	11.12	38	0	0	0.21	90.31	X														
163	Carnosol	C20H26O4	329.1745	12.23	37.9	0.0	0	-4.12	94.29								X					X		
	**LIGNANS**																							
164	Sesaminol/Episesaminol I	C20H18O7	369.0979	1.71	38.7	0.0	0	-0.31	93.76							X	X		X		X		X	X
165	Enterolactone	C18H18O4	297.1158	11.37	34.4	0.0	0	8.50	81.44								X							
166	1-Acetoxypinoresinol	C22H24O8	415.1365	3.57	35.6	5.3	135.0430 (100%)	-8.05	81.86								X					X		
167	Schisantherin A	C30H32O9	535.2024	4.06	35.9	0.0	0	9.34	89.60									X						
168	Sesaminol/Episesaminol II	C20H18O7	369.0945	4.40	34.5	0.0	0	-9.29	82.79							X					X		X	
169	Dimethylmatairesinol/Gomisin M2	C22H26O6	385.1621	5.29	35.7	0.0	0	-9.24	88.57									X						
	**STILBENES**																							
170	Viniferin/Pallidol	C28H22O6	453.1352	4.64	36.1	0.0	0	1.84	82.55									X						
171	Resveratrol 3-O-glucoside	C20H22O8	389.1230	5.40	39.7	10.9	265.0731 (5.83%)	-2.99	91.02							X	X	X			X	X	X	
172	Piceatannol	C14H12O4	243.0648	6.08	37.9	0.0	0	-5.91	96.22		X			X										X

m/z = mass/charge. Bold represent reference standards.
